# Slow-release nitrogen fertilizers enhance growth, yield, NUE in wheat crop and reduce nitrogen losses under an arid environment

**DOI:** 10.1007/s11356-021-13700-4

**Published:** 2021-04-09

**Authors:** Iqra Ghafoor, Muhammad Habib-ur-Rahman, Muqarrab Ali, Muhammad Afzal, Wazir Ahmed, Thomas Gaiser, Abdul Ghaffar

**Affiliations:** 1 Department of Agronomy, MNS University of Agriculture Multan, Multan, Punjab Pakistan; 2 Crop Science Group, Institute of Crop Science and Resource Conservation (INRES), University of Bonn, Bonn, Germany; 3 AgWeatherNet Program, Washington State University, Prosser, WA USA; 4 Legume Research Unit, Molecular Biology Lab, Department of Plant Production, King Saud University, Riyadh, Saudi Arabia; 5 Department of soil science, MNS University of Agriculture Multan, Multan, Punjab Pakistan

**Keywords:** Partial factor productivity, Partial nutrient uptake, Nitrate leaching, Adaptation for climate change

## Abstract

Higher demands of food led to higher nitrogen application to promote cropping intensification and produce more which may have negative effects on the environment and lead to pollution. While sustainable wheat production is under threat due to low soil fertility and organic matter due to nutrient degradation at high temperatures in the region. The current research explores the effects of different types of coated urea fertilizers and their rates on wheat crop under arid climatic conditions of Pakistan. Enhancing nitrogen use efficiency by using eco-friendly coated urea products could benefit growers and reduce environmental negative effects. A trial treatment included N rates (130, 117, 104, and 94 kg ha^-1^) and coated urea sources (neem coated, sulfur coated, bioactive sulfur coated) applied with equal quantity following split application method at sowing, 20 and 60 days after sowing (DAS). The research was arranged in a split-plot design with randomized complete block design had three replicates. Data revealed that bioactive sulfur coated urea with the application of 130 kg N ha^-1^ increased chlorophyll contents 55.0 (unit value), net leaf photosynthetic rate (12.51 μmol CO_2_ m^-2^ s^-1^), and leaf area index (5.67) significantly. Furthermore, research elucidates that bioactive sulfur urea with the same N increased partial factor productivity (43.85 Kg grain Kg^-1^ N supplied), nitrogen harvest index (NHI) 64.70%, and partial nutrient balance (1.41 Kg grain N content Kg^-1^ N supplied). The neem-coated and sulfur-coated fertilizers also showed better results than monotypic urea. The wheat growth and phenology significantly improved by using coated fertilizers. The crop reached maturity earlier with the application of bioactive sulfur-coated urea than others. Maximum total dry matter 14402 (kg ha^-1^) recorded with 130 kg N ha^-1^application. Higher 1000-grain weight (33.66 g), more number of grains per spike (53.67), grain yield (4457 kg ha^-1^), and harvest index (34.29%) were obtained with optimum N application 130 kg ha^-1^ (recommended). There is a significant correlation observed for growth, yield, and physiological parameters with N in the soil while nitrogen-related indices are also positively correlated. The major problem of groundwater contamination with nitrate leaching is also reduced by using coated fertilizers. Minimum nitrate concentration (7.37 and 8.77 kg ha^-1^) was observed with the application of bioactive sulfur-coated and sulfur-coated urea with lower N (94 kg ha^-1^), respectively. The bioactive sulfur-coated urea with the application of 130 kg N ha^-1^ showed maximum phosphorus 5.45 mg kg^-1^ and potassium 100.67 mg kg^-1^ in the soil. Maximum nitrogen uptake (88.20 kg ha^-1^) is showed by bioactive sulfur coated urea with 130 kg N ha^-1^ application. The total available NPK concentrations in soil showed a significant correlation with physiological attributes; grain yield; harvest index; and nitrogen use efficiency components, i.e., partial factor productivity, partial nutrient balance, and nitrogen harvest index. This research reveals that coating urea with secondary nutrients, neem oil, and microbes are highly effective techniques for enhancing fertilizer use efficiency and wheat production in calcareous soils and reduced N losses under arid environments.

## Introduction

Feeding the ever-increasing world population requires more attention for effective and precise use of limited resources like fertilizers. Wheat crop is among the top in cereals to provide the food. In the mid-term (2050) wheat requirement will increase up to 840 million tons from its current production rate of 642 million tons (Sharma et al. 2015). The production is declined due to various factors like drought stress, late sowing, low-quality seeds, climate variability, and insect pests, but judicial use of fertilizers is also important for wheat production for food security and the environment (Hochman and Horan 2018; Rahman et al. 2018). Nitrogen (N) is a major element for increasing wheat production. The nitrogen use efficiency (NUE) indicates the ability of plants for N uptake and change available N into the economic part (Sher et al. 2019). The NUE is noted under 50% for cereal crops comprising wheat because grain crops required maximum nitrogen for higher economic yield (Rahimizadeh et al. 2010). The N application is sensitive and must be relating to the crop’s need (Slafer and Savin 2018; Rahman et al. 2019). Nitrate is a common form of N and is found in the cell vacuole and reduced in the cytosol by nitrate and nitrite reductase activity. These machinery contained chlorophyll that is important for photosynthesis. There are two important considerations about N uptake stated when N is readily available in the soil solution and plant required N at that time (Lin et al. 2017). Excessive nitrogen causes environmental pollution and economic losses (Rahman et al. 2020). The injudicious use of N fertilizers causes lodging in crops and reduced economic yield. Crusciol et al. (2019) observed that proper application methods reduced N volatilization and the number of seeds per spike. The optimum N fertilizers rate proved to increase thousand-grain weight, protein contents, NUE, and wheat yield (Zhang et al. 2015). To reduce the N losses and increase the yield, the 4R principle (right time, the right amount, right source, and right place) is suggested to adopt for fertilizers (Flis 2017). Further, it is also revealed 50 years ago by the law of diminishing return that ever increased fertilizer application will not be a good technique in enhancing crop’s yield (Pleijel et al. 2019).

The NUE can be enhanced by using slow-release nitrogenous fertilizers. The N application except nitrate is the main problem when applied to promote plant growth and development. The N losses are a major threat to environmental pollution that causes health issues. About 50% of ammonia volatilized globally from the agriculture sector (Conijn et al. 2018). The ammonia volatilization from urea fertilizer recorded 0–65% depends on environmental and soil characteristics (Bowles et al. 2018; Bishop and Manning 2010). Nitrate water pollution causes serious health issues in humans and animals. Higher N application than the crop requirements is the possible cause of low NUE and N losses in the soil. The N losses lead to a reduction in NUE of the crops. The efforts to increase NUE is a major concern in the agriculture sector for decades (Dobermann 2005). The various techniques for enhancing NUE have been recognized (Anas et al. 2020). The concept of slow-release nitrogenous fertilizers is likely to adopt to reduce environmental and water pollution.

The slow-release fertilizers have semi-permeable layers of different essential oils, secondary and primary nutrients which controlled the granular water solubility by slowing the process of hydrolysis of water-soluble fertilizers (Trenkel 2010; Naz and Sulaiman 2016). Sulfur-coated urea (SCU) increased wheat growth and development compared with monotypic urea. The wheat crop has a positive correlation between “S” and “N” elements (Klikocka et al. 2017). The “S” element is used as a secondary element, fungicide, and has acidic properties that neutralized the soil alkalinity (Azeem et al. 2014). Therefore the excessive application of N without coating material “S” causes maximum N leaching and decreases NUE (Shivay et al. 2016). The slow-release neem-coated urea (NCU) has nitrification inhibitor characters and enhanced NUE and yield. Basically, the neem oil consists of de-acetyl, azadirachtin, epinimbin, melicians, and salanin components which presented treatment-based nitrification inhibition method (Khandey et al. 2017). Therefore, the judicial use of nitrogen fertilizers and sources decline losses, increased NUE, and crop production. The ecological-friendly fertilizers products are produced by degradable microorganism’s material coating that increased the diffusion duration of fertilizer granular by slow release procedure (Conrad 2000; Chen et al. 2018). Nitrogen application is predicted to increase three times by 2050 for grain crop production in developing countries. There is limited research available about the effects of coated urea on wheat under arid climatic conditions. There is a need to test their adaptability under arid climatic conditions to reduce the N losses. The current research is planned to enhance wheat growth and development and sustainable soil management and improve NUEs through different coated urea fertilizers (neem, sulfur (S), and bioactive S-coated urea (BSCU)) under arid environment. The objectives were to evaluate the N sources and rates best for wheat production, higher NUE, and lower N losses under arid climatic conditions to ensure food security.

## Materials and methods

### Experimental site and environmental conditions

**Fig. 1:**
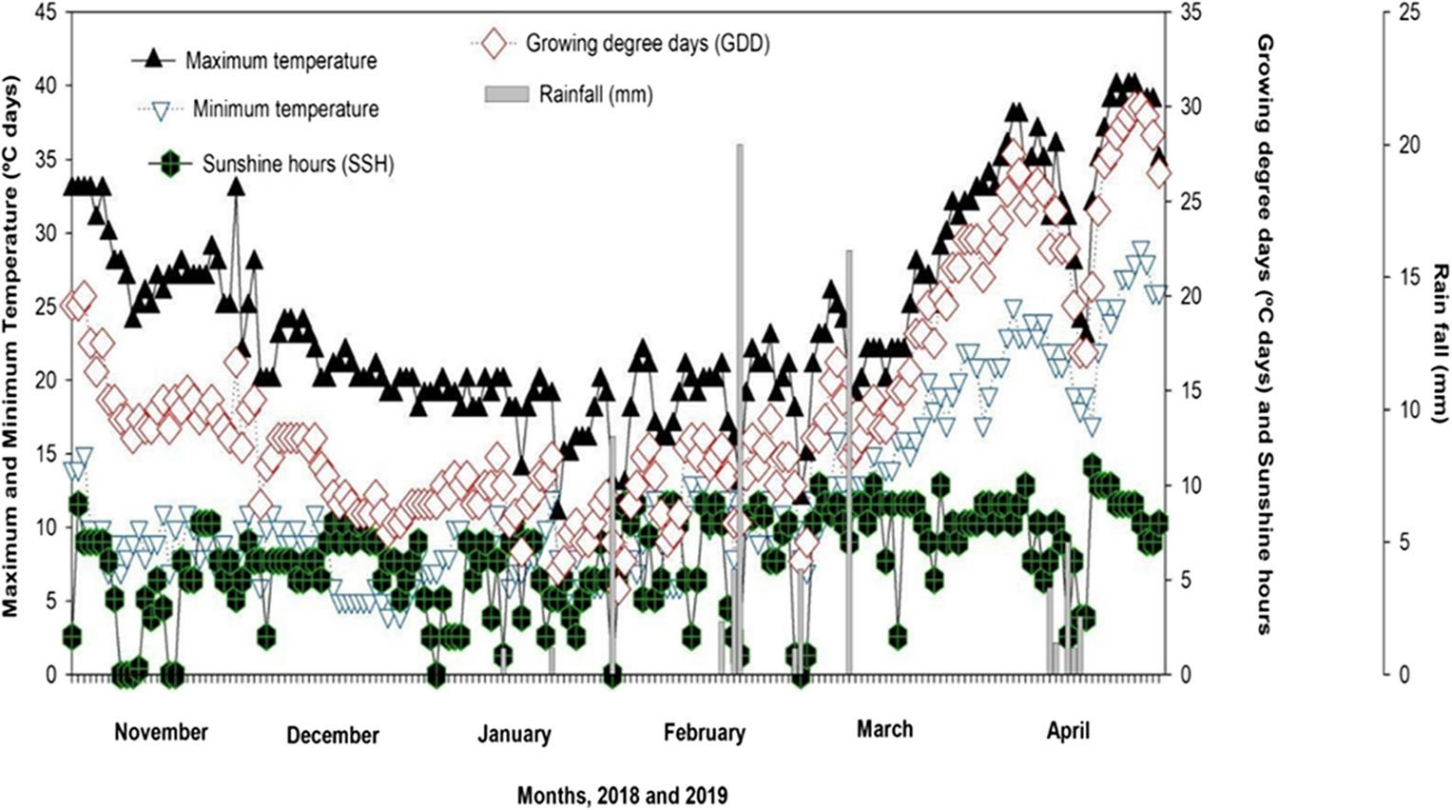
Daily weather variables (max. and mini. air temperature, sunshine hours, rainfall, and GDDs calculated on a thresh hold a temperature of 4 °C) during wheat crop growing seasons

A field experiment was performed during 2018-2019 in arid climatic conditions of southern Punjab, Pakistan (30.1598° N, 71.4502° E and altitude of 129 m). Multan area was classified as arid with the occurrence of cold winters, extreme temperature fluctuations, windstorms, and erratic rainfall patterns. In the experiment area, the diurnal variations and fluctuations were observed where regular mean air temperature throughout the winter and summer season ranged from 4 to 23 °C and 26 to 49 °C, respectively. An extremely flexible rainfall occurs during the monsoon season (July–August), but an amount of 66 mm rainfall occurred during crop growing season, which did not fulfill the crop water’s demand. Daily temperature (Tmin, Tmax), sunshine hours, and rainfall data were being recorded in an automatic weather station (AWS) installed 16 m away from the experimental site while growing degree days (GDDs) above a particular threshold temperature computed for the wheat crop (Fig. 1).

The research zone is situated near River Chenab (running whole year) in district Muzaffar Garh. The texture and color of well-drained soil are loam with saturation 38% and brown, respectively. Due to high temperature, the organic matter of soil varied from 0.51 to 0.68%. The majority of the soils have a minimum available P and K 8.10 and 225 mg kg^-1^, respectively, but the soil is alkaline in nature with higher pH (7.9–8.8). While soil is deficient in total N, availability ranged 0.014 to 0.027% in upper layers (0–15 and 15–30 cm), respectively. The soil has also higher (1.14–7.41 mS cm^-1^) electrical conductivity (EC) in the depths of 0–15 and 15–30 cm. According to Soil Survey of Punjab, Pakistan, this research zone has Shujabad soil series with minor components (30%) of Miani-Shujabad, (40%), Miani, and (15%) and Sultanpur soil series, respectively. Initial soil physiochemical properties before the start of the experiment are mentioned in Table 1.

**Table 1:** Physiochemical soil analysis before the start of the experiment

Parameters	Unit	Value
pH		8.8
EC	mS cm^-1^	0.7
Saturation	%	38
Available P	(mg kg^-1^)	8.10
Available K	(mg kg^-1^)	225
Total N	%	0.027
Organic matter	%	0.68

### Experimental design, treatment structure, and crop management

The research experiment was conducted to assess the performance and effect of different coated urea fertilizers on winter season wheat crop growth and development and nitrogen use efficiency under various N-based treatment combinations. Neem-coated urea and simple urea were provided by Engro Fertilizers Limited Pakistan, while bioactive S-coated urea (BSCU) (Thiobacillus) is a product of First Microbial Bio-Tech. The S-coated urea was got from Adfert UAE. The current research experiment consisted of 16 treatments, comprising of four nitrogen sources (simple urea, neem coated, sulfur coated, and bioactive sulfur coated urea’s), with four N rates (130 (recommended dose), 117, 104, and 94 kg ha^-1^) having three replicates (4×4×3=48 experimental units) tested in a randomized complete block design (RCBD) with split plot arrangement. The tractor-mounted plow was used to plow the soil 3 times followed by land leveling then assigned experimental units. The experimental units and blocks were detached by 100 cm from both sides from preventing N losses and border effects. The full basal dose of Single Super Phosphate (SSP) and Sulphate of Potash (SOP) was applied (114 kg ha^-1^ and 62 kg ha^-1^), respectively, according to the pre-soil analysis report. All urea fertilizers were applied in 3 equal splits (at sowing time, 20 and 60 DAS). The well-grown wheat cultivar which is adopted at farmer field was used (Ayub Agricultural Research Institute Faisalabad (AARI), Pakistan). The research experiment was sown by drill method with seeds rate of 60 kg ha^-1^ and maintained line to line recommended distance of 22 cm. The recommended irrigation (250 mm) was applied before sowing, tillering initiation, and anthesis stages to meet the wheat crop water requirements to grow under a non-stress environment. All other agronomic mechanical and chemical-related control of weeds and insect’s operations were uniformly adopted for all plots to provide the best micro condition for vigorous plant growth and development.

### Soil analysis techniques

EC was evaluated by using the saturation extract procedure available in US Salinity Lab Staff 1954. Available soil phosphorus was analyzed by Olsen procedure (Olsen et al. 1954). The available soil potassium was measured by using the flame photometer method (US Salinity Lab Staff 1954). The available organic matter and total N (%) were estimated by Walkley-Black method (Ryan et al. 2013) and the Kjeldahl method (Bremner 1960), respectively at 20, 60, and 120 DAS. Soil nitrate analysis was done by using chromotropic acid procedure (Cataldo et al. 1975).

### Plant NPK estimation procedures and protocols

The plant N contents were measured at 20, 60, and 120 DAS by following the standard procedure mentioned by Jones Jr (1991). The plant-dried sample (1.0 g) was put in a digestion tube. Then mix 15 ml of concentrated H_2_SO_4_ and 1 g digestion mixture (K_2_SO_4_+CuSO_4_ @ 9:1), and heat the tubes at 450 °C for 2 h in a digestion block. The solution color was transparent to yellowish or green after heating clearly seen in digestion tubes. In a distillate unit, required volume for the distillation process is made. Then add material in 4% boric acid (25 ml) receiver. After this, put few indicator drops and the purple color was showed and then altered into golden yellow during the distillation procedure. Then collected distillates were titrated by adding 0.1 N H_2_SO_4_ and the purple color endpoint was showed again from golden yellow shade (Jones Jr 1991).

The P% in the plant was estimated by the following spectrophotometer and using the spectrophotometric vanadium-phosphomolybdate procedure. The standard of P samples was run on the spectrophotometer. After this, the yellow color procedure, P concentration in plant samples was estimated. The digested plant samples, distill water, and coloring reagent were added to a flask. Then the flask was kept at room temperature for about 30–35 min. It showed color over time. Then run the P% in plant samples determined by using a spectrophotometer at 420 nm (Olsen et al. 1954). The K concentration was measured by flame photometer procedure introduced by Chapman and Parker (1961).

### N determination in grain

The wheat grain 0.1 g was added to a digestion tube and 5 ml of H_2_SO_4_ concentration was put in it. Then tubes were incubated at 25 °C temperature for 12 h cautiously. Then mix 1 ml of (H_2_O_2_) (34%) in the digestion tubes. After this retain tubes under 350 °C and wait for flame production. The heating time was set for 30 min. Then chill the digestion tubes after reheating process. Then mixed 1 ml of H_2_O_2_ and put tubes again in the digestion block. This procedure was repeated several times until colorless material was shown. After this, material was kept in the flask (50 ml) and extract volume was arranged very carefully. After this, the filtrate nitrogen content of grains was obtained by using Kjeldahl’s method.

### Procedure and protocols for growth and yield parameters

Measurements of yield and yield-related attributes, i.e., plant height, seeds per spike, number of tillers m^-2^, total dry matter (kg ha^-1^), average seeds weight per spike, grain yield (kg ha^-1^), and harvest index, were measured from each experimental unit. Data on physiological parameters such as chlorophyll contents% and net photosynthetic rate were measured by portable chlorophyll meter (SPAD-502, Konica Minolta, Europe) and portable photosynthesis system (CIRAS-3, PP Systems-Hitchin, United Kingdom), respectively. Wheat plants were harvested at 15 days’ interval to assess the fresh biomass and then dry matter. The row plant was evocative of a whole unit and leaving unit borders. The leaves and stem fresh weights were calculated by electrical weight balance. Then various components like leaf and stem were oven-dried for 48 h at 70 °C and after this dry weight was estimated. Leaf area was estimated with the help of a leaf area meter (Model, CI-202, CID Bio_,_ Science, Inc. 1554 NE 3^rd^ Ave Camas, WA98607). Leaf area index (LAI) was measured by dividing leaf area by land area (Hunt 1978). Leaf area duration (LAD) was calculated by using the formula (LAI_1_+LAI_2_) x (T_2_-T_1_)/2 (Hunt 1978), where LAI_1_ and LAI_2_ are leaf area indexes at times T_1_ and T_2_ correspondingly. Crop growth rate (CGR) was estimated as (W_2_-W_1_)/(T_2_-T_1_) (Hunt 1978), whereas W_1_ and W_2_ are the dry weights collected at time T_1_ and T_2_ respectively. At maturity, 20 wheat plants from each experimental unit were collected, and different yield components were computed. The final yield and biomass were assessed from the whole experimental unit separately and on basis of dry biomass accumulation converted into kg ha^-1^.

### Measuring NUEs and Plant N uptake

Partial factor productivity (PFP) (Kg grain Kg^-1^ N supplied) is the ratio between grain yields (kg) per kg to N applied (Nielsen 2006). Partial nutrient balance (PNB) (Kg grain N content Kg^-1^ N supplied) is the ratio between total N contents (kg) per kg of N application (Snyder et al. 2007; Hawkesford 2012). N harvest index (NHI%) is determined by dividing total grain N (Ng) by total N (Nt) that was calculated by multiplying plants dry weight of N contents and adding over total parts of plant consumption (Rahimizadeh et al. 2010). The total N uptake by plants also determined by using wheat plants N concentration against total N application. The plant N uptake (Kg ha^-1^) is calculated by following the formula presented by Ali et al. (2020).

1 $$ \mathrm{Plant}\ \mathrm{N}\ \mathrm{uptake}\ \left(\frac{\mathrm{kg}}{\mathrm{ha}}\right)=\frac{\mathrm{Total}\ \mathrm{N}\%\mathrm{in}\ \mathrm{plants}\ }{100}\times \mathrm{Total}\ \mathrm{dry}\ \mathrm{matter}\ \left(\frac{\mathrm{kg}}{\mathrm{ha}}\right) $$

### Statistical analysis

Statistical analysis was performed using the R statistical software (R version 3.6.1, https://www.r-project.org) by adopting Fisher’s two-way factorial ANOVA and Tukey’s HSD test at a 5% probability level to determine the means contrasts among treatments. We also initially checked the data for homogeneity of variance and normality using Levene’s test and Shapiro–Wilk test, respectively. The correlation and regression analysis were done for obtained clear results between assorted treatments (Steel et al. 1997).

## Results

### Phenology and wheat development

**Fig. 2:**
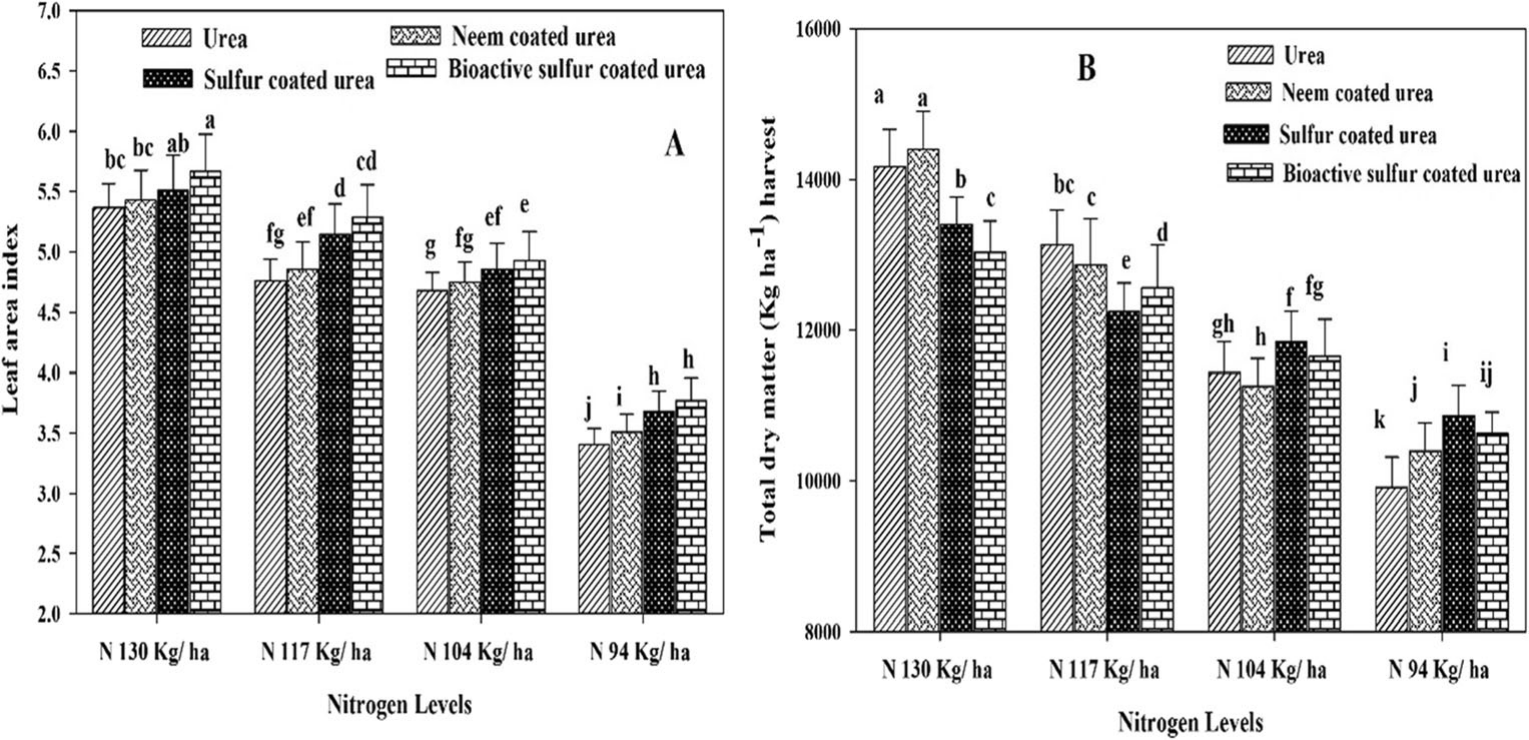
Interactive effect of different N levels and slow-release urea fertilizers on peak LAI (**a**) at 90 DAS and TDM at harvest (**b**) of wheat under arid environmental conditions

**Fig. 3:**
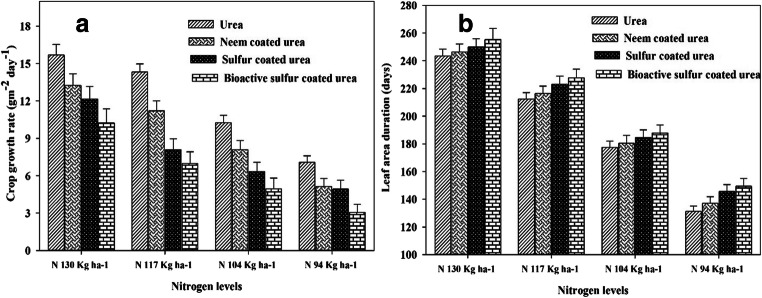
Interactive effect of different N levels and slow-release urea fertilizers on peak (75 DAS) wheat CGR (gm^-2^ day^-1^) (a) and LAD (days) (**b**) under arid environmental conditions

There is no significant effect of N and different coated urea fertilizers observed for seedling emergence of wheat crop. Crop reached to anthesis stage late (112 days) when applied maximum N (130 kg ha^-1^) as urea source followed by NCU and both were found statistically similar. while crop reached anthesis earlier (96 days) with the application of lower N (94 kg ha^-1^) with NCU and SCU (N sources). Similar results, where crop reached maturity late (144 days) with the application of simple urea (130 kg N ha^-1^) and followed by NCU and SCU at same N application rate. Early crop maturity was observed (121 days) with lower N application (94 kg ha^-1^) and BSCU, but it was statistically similar with NCU with the same N application (Table 2). The maximum LAI (5.67) was attained at 90 DAS with the application of BSCU at 130 kg N ha^-1^ (Fig.2 A), while minimum LAI was recorded where neem-coated urea is applied with the lowest N (94 kg ha^-1^) application. The N 130 kg ha^-1^ BSCU and N 94 kg ha^-1^ simple urea showed maximum and minimum LADs, respectively, than other treatments (Fig. 3b). Maximum crop growth rate (gm^-2^ day^-1^) recorded with the application 130 kg N ha^-1^ urea at 75 DAS (days after sowing) than other sources and rates. The minimum crop growth rate is recorded with the application of lower N (94 kg ha^-1^) and BSCU while it was at par with 104 kg N ha^-1^ BSCU, N 94 kg ha^-1^ NCU, SCU, and simple urea as well (Fig. 3a).

**Table 2:** Effect of different N levels and slow-release urea fertilizers on phenological phases and stages for wheat crop under arid climatic conditions

Treatments	Days to emergence	Days to anthesis	Days to physiological maturity
UreaN_0_	12 a	112 a	144 a
UreaN1	13 a	105 bcd	132 cd
UreaN2	12 a	101 def	128 def
UreaN3	13 a	97 ef	122 gh
NCUN_0_	10 a	112 a	142 ab
NCUN1	12 a	103 cde	129 de
NCUN2	14 a	100 def	126 efgh
NCUN3	12 a	97 f	121 h
SCUN_0_	13 a	109 ab	139 ab
SCUN1	13 a	101 def	127 efg
SCUN2	12 a	98 ef	123 fgh
SCUN3	11 a	98 ef	122 gh
BSCUN_0_	12 a	107 abc	137 bc
BSCUN1	11 a	97 ef	125 efgh
BSCUN2	13 a	100 def	126 efgh
BSCUN3	10 a	96 f	121 h
Interaction Sources x N levels	Non-significant	Significant (2.35)	Significant (2.61)

*NCU* neem-coated urea, *SCU* sulfur-coated urea, *BSCU* bioactive sulfur-coated urea

*N*_*0*_= 130 kgha^-1^; *N1*= 117 kgha^-1^; *N2*= 104 kgha^-1^; *N3*= 94 kgha^-1^

### Agronomic and yield-related attributes of a wheat crop

**Fig. 4:**
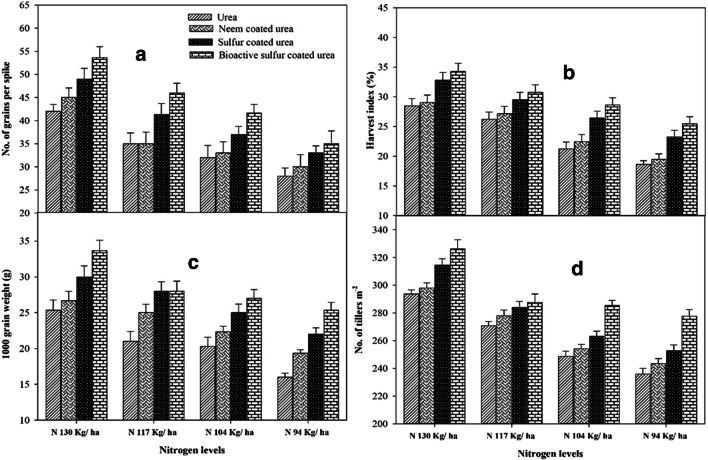
Interactive effect of different N levels and slow-release urea fertilizers on number of grains per spike (a), H.I. (%) (b), 1000 grain weight (g) (c), and number of tillers m^-2^ (d) under arid environmental condition

The coated urea and N addition enhanced soil fertility and resulted in yield improvement and associated parameters (number of tillers, grains per spike, grains weight), and final dry matter. High N rate (130 kg ha^-1^) with BSCU produced significantly more yield attributes like the number of tillers (326 m^-2^) and finally yield than SCU, NCU, and simple urea with the same amount of N applied. A minimum number of tillers was produced with lower N application with simple urea application. Similarly, the recommended N (130 kg ha^-1^) with BSCU produced higher grains per spike (54) than the minimum ones (28) where lower N (94 kg ha^-1^) was applied in the form of simple urea. Maximum and minimum thousand-grain weight of 33.66 g and 16 g were recorded in experimental units where N 130 and 94 kg ha^-1^ BSCU and urea were applied, respectively (Fig. 4).

**Fig. 5:**
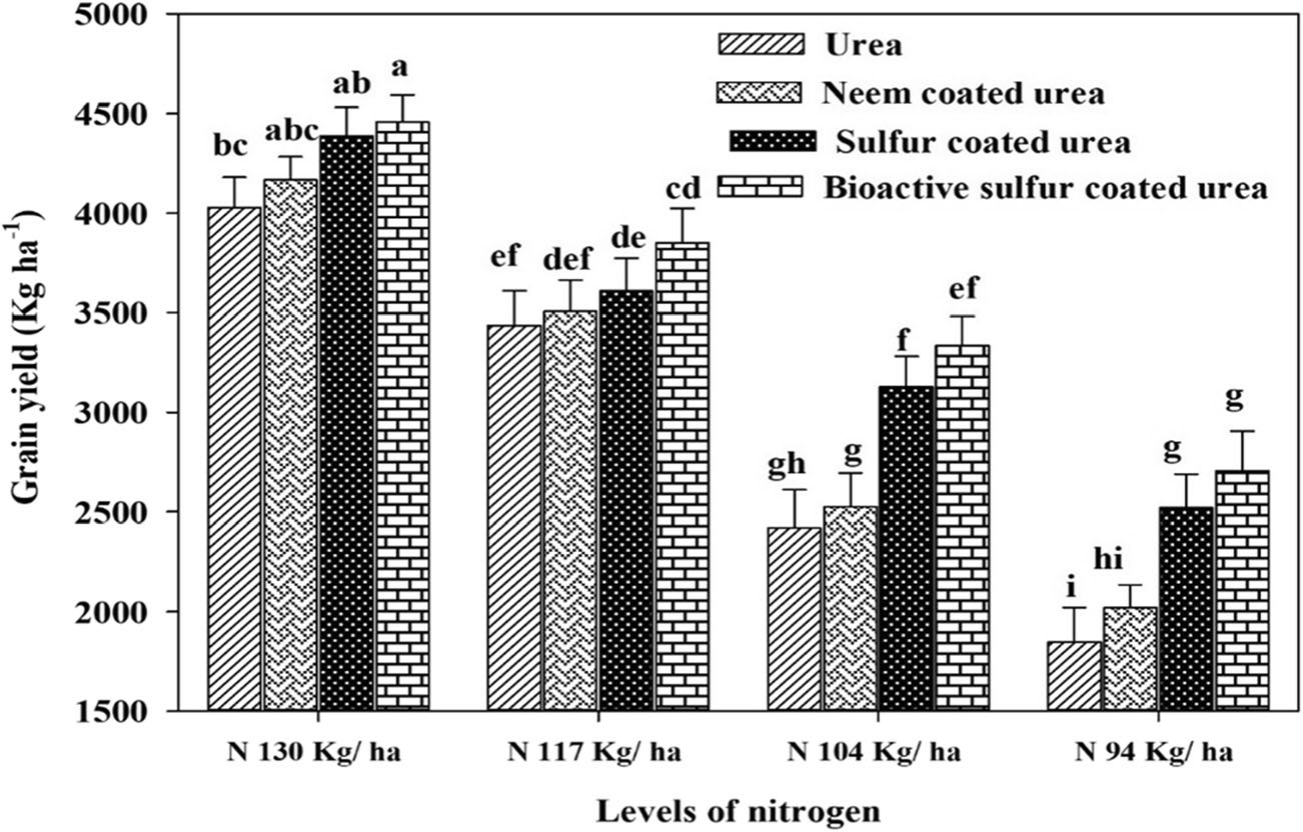
Interactive effect of different N levels and slow-release nitrogen fertilizers on grain yield (Kg ha^-1^) of wheat crop under arid environmental conditions

Results demonstrated that the final TDM (harvest) was affected by the N application with different urea sources together. Maximum TDM (14177 kg ha^-1^) produced where high N applied in the form of simple urea and it was statistically the same for NCU (with the same N), while the lower dry matter produced (9916 kg ha^-1^) with lower N urea than NCU, SCU, and BSCU with equal N application (Fig. 2b). There is high HI recorded (34.29%) by using high N BSCU while the lowest H.I. (18.61%) was observed by N 94 kg ha^-1^ simple urea application (Fig.4). Remarkably, 130 kg N ha^-1^ BSCU performed well as compared with other treatments. Results revealed a 10% improvement in grain yield with the application of 130 kg N ha^-1^ BSCU than simple urea source. The coated fertilizers SCU and NCU with an N rate of 130 kg ha^-1^ showed an increase of 8.19 and 3.34% vs simple urea, respectively. Furthermore, previous results of yield attributes showed the minimum grain yield (1847 kg ha^-1^) was produced with the application of lower N in the form of simple urea than other sources (Fig. 5).

**Fig. 6:**
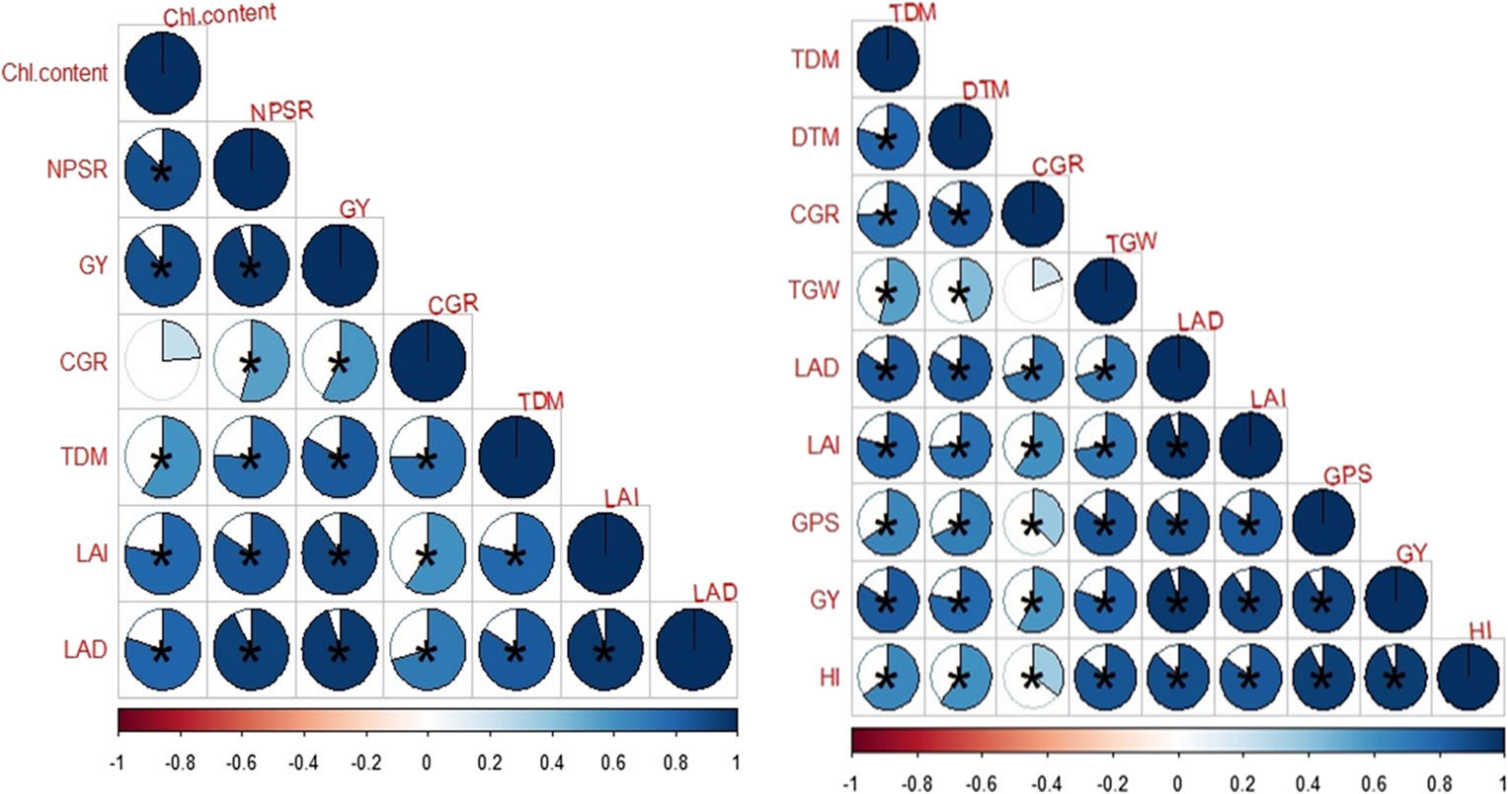
Correlation of different growth parameters with yield and yield-related attributes at different N sources and rates. The areas of circles show the absolute value of corresponding correlation coefficients tested at *0.01 significance level. Dark blue and light blue color showed the higher and lower values. Star (*) showed the significance and non-star showed non-significance. *TDM* total dry matter, *DTM* days to maturity, *CGR* crop growth rate, *TGW* total grain weight, *LAD* leaf area duration, *LAI* leaf area index, *GY* grain yield, *HI* harvest index, *GPS* grains per spike, *NPSR* net photosynthetic rate, and *Chl. contents* chlorophyll contents

Growth parameters (LAI, TDM) were also affected by N increment and coating urea, where high LAI and TDM are produced with the application of high N than lower N application. The results of the current research showed a positive relationship between coated urea N rates and growth attributes like leaf area, TDM, and grain production (Fig. 6). The TDM enhanced with the passage of time and conquering higher LAI due to the maximum availability of N in the soil. Maximum LAD increased crop vegetative growth and TDM, because sufficient availability of N in soil stayed leaves green for a maximum period resulting in higher CGR.

### Crop physiological parameters

**Fig. 7:**
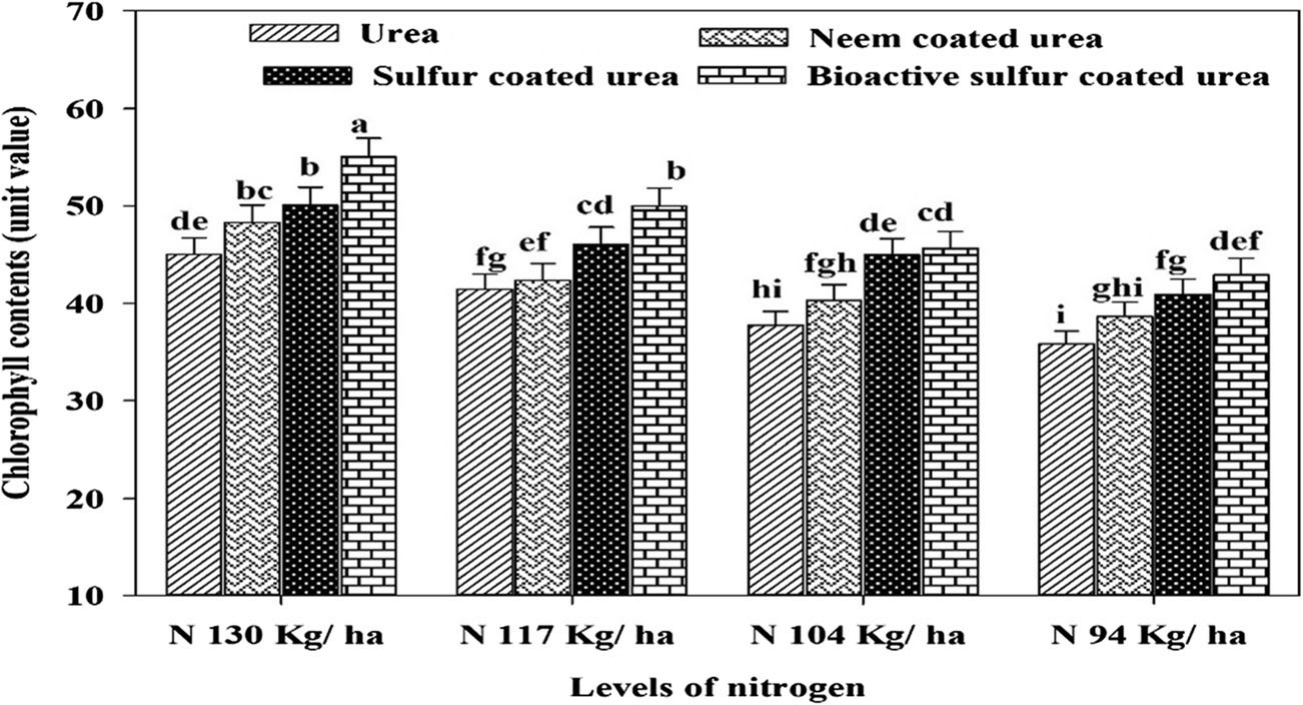
Effect of different N levels and slow-release urea fertilizers on chlorophyll contents (unit value) at 75 DAS under arid environmental conditions

**Fig. 8:**
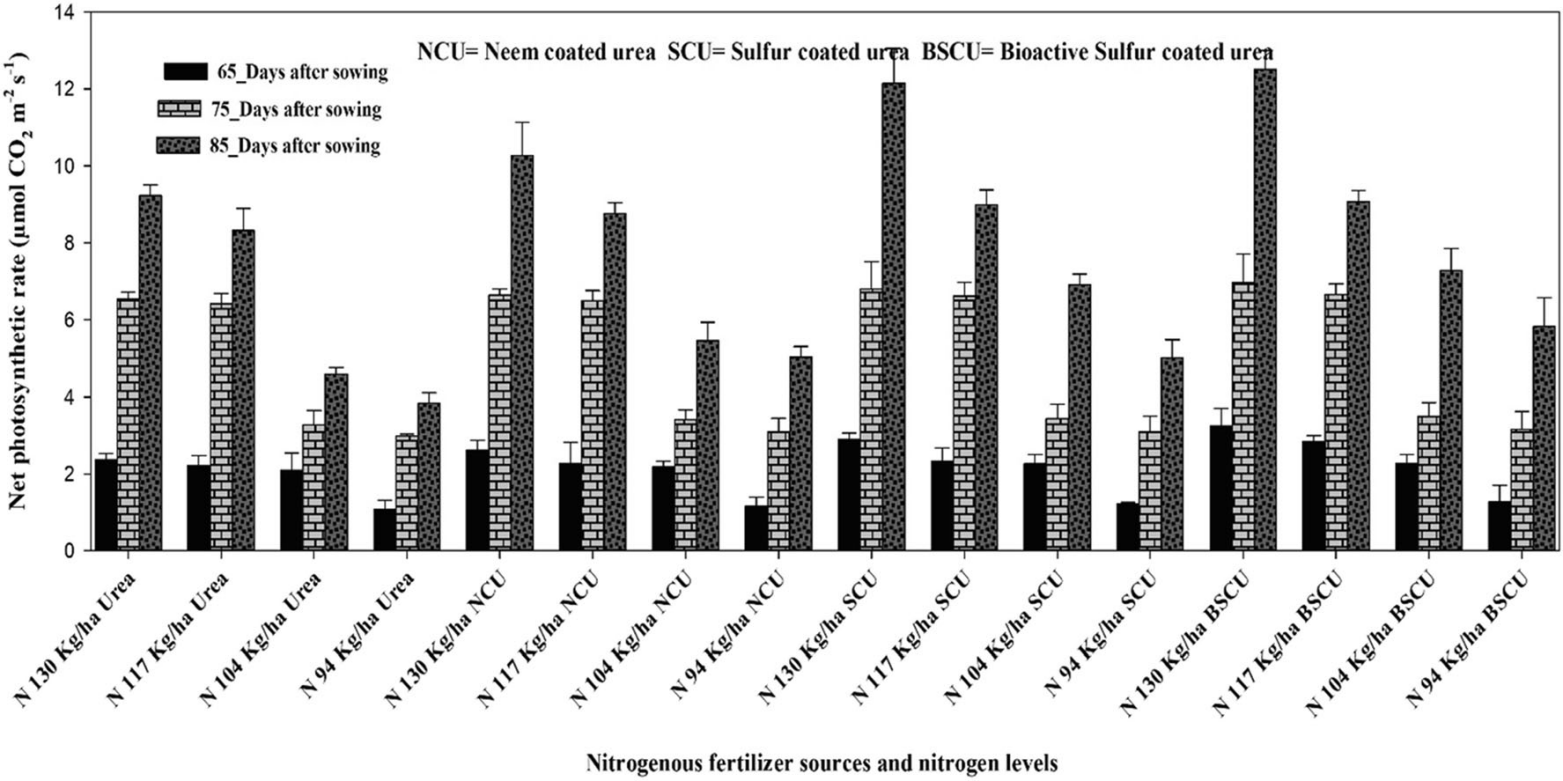
Interactive effect of different N levels and slow-release urea fertilizers on time series data (65, 75, and 85 DAS) of leaf net photosynthetic rate (μmol CO_2_ m^-2^ s^-1^) in wheat crop under arid environmental conditions

Coated fertilizers significantly improve the chlorophyll contents than monotypic urea. A higher value of chlorophyll contents (55.0-unit value) was produced with 130 kg N ha^-1^ BSCU while the lowest (35.86-unit value) recorded where lower N applied in the form of simple urea (Fig.7). The research data revealed that the N 94 kg ha^-1^ BSCU application showed an increase of 4.90, 10.00, and 16.51-unit values than SCU, NCU, and urea, respectively. Almost, similar results as previously also recorded for physiological parameters like higher net leaf photosynthetic rate recorded (12.51 μmol CO_2_ m^-2^ s^-1^) for 130 kg N ha^-1^ BSCU source than others. The N 130 kg ha^-1^ BSCU revealed an increase vs SCU, NCU, and urea as 2.87, 17.91, and 26.22%, respectively, with similar N rates. The N level 94 kg ha^-1^ urea showed a minimum net photosynthetic rate of 3.83 (μmol CO_2_ m^-2^ s^-1^). The N showed a positive correlation with chlorophyll contents and net photosynthetic rate (μmol CO_2_ m^-2^ s^-1^). The N improved the photosynthetic pigments and chlorophyll contents of wheat which lead to an improving net photosynthetic rate (Fig. 8). The higher net photosynthetic rate (μmol CO_2_ m^-2^ s^-1^) resulted in higher growth and dry matter and ultimately more grain production due to improvement in source and sink relationship (Fig. 6 ).

### NUE and components of wheat crop under arid environment

**Fig. 9:**
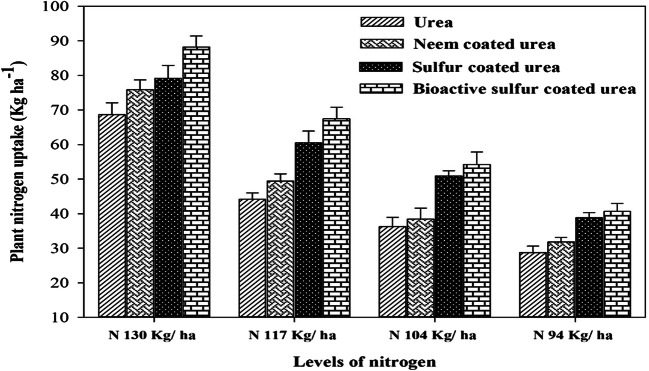
Interactive effect of different N levels and slow-release urea fertilizers on plant nitrogen uptake (Kg ha^-1^) under arid environmental conditions

**Fig. 10:**
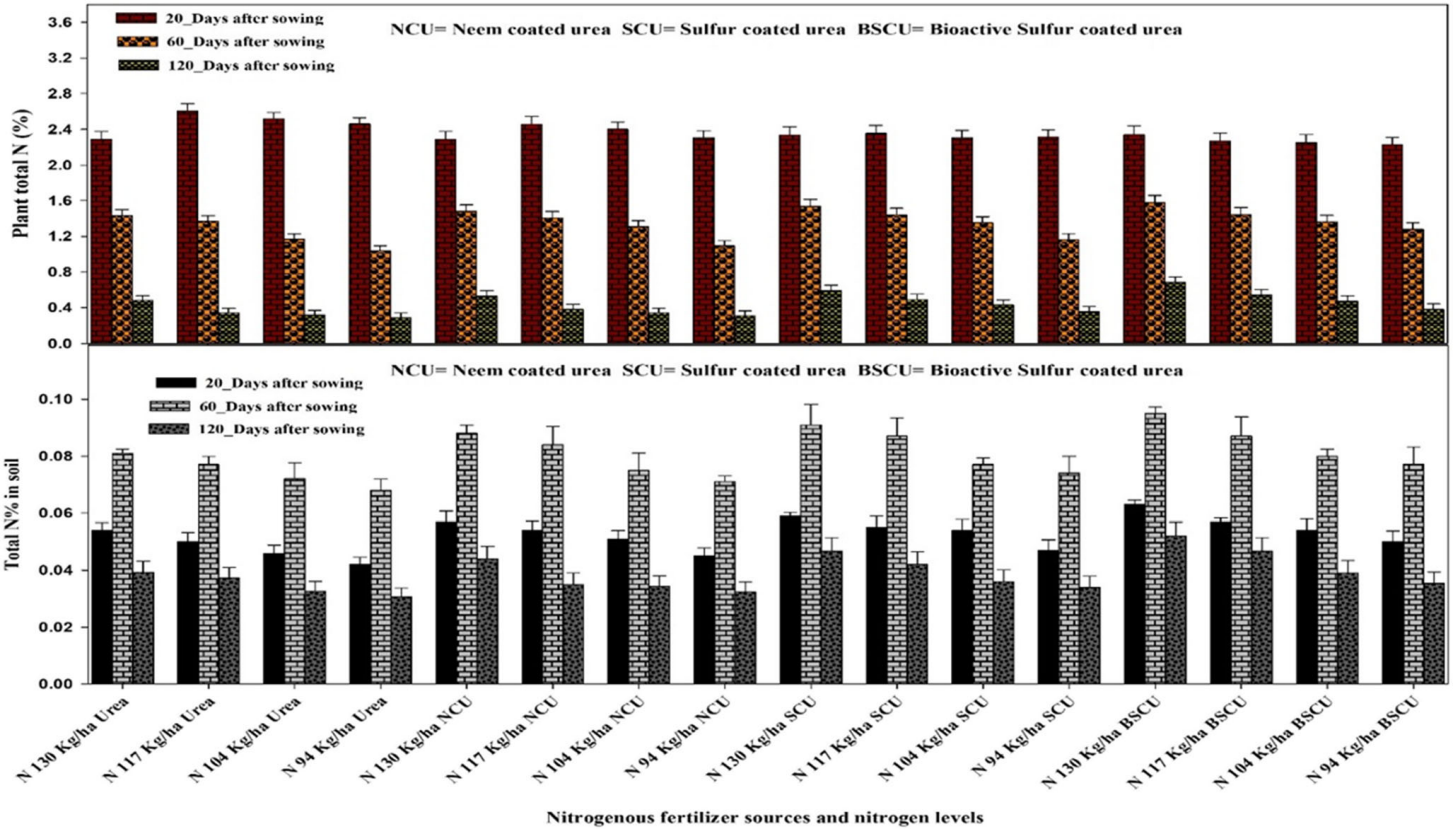
Temporal interactive effect of different N levels and slow-release urea fertilizers on total N in soil (%) and plant total N (%) in plants under arid environmental conditions

**Fig. 11:**
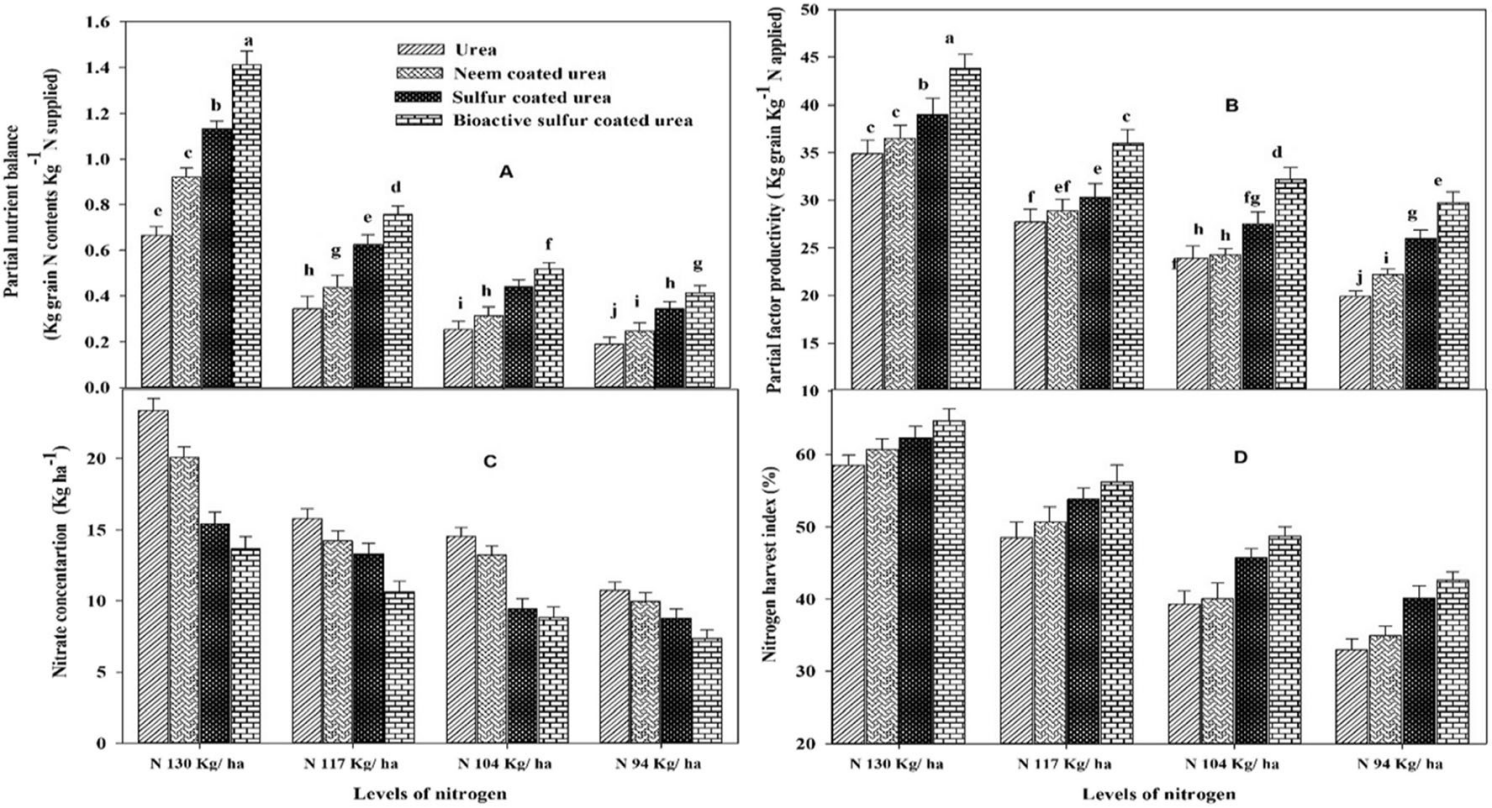
Effect of different N levels and slow-release urea fertilizers on (**a**) partial nutrient balance (Kg grain N content Kg^-1^ N supplied), (**b**) partial factor productivity (Kg grain Kg^-1^ N applied), (**c**) Nitrate concentration (Kg ha^-1^), and (**d**) nitrogen harvest index (%) of wheat under arid environmental conditions

The NUE was computed through its components like PFP (Kg grain Kg^-1^ N supplied) and PNB (Kg grain N content Kg^-1^ N supplied). Treatments with coated urea fertilizers revealed a higher plant N uptake than simple urea, like maximum N plant uptake (89 kg ha^-1^) recorded in the experimental plots where 130 kg N ha^-1^ BSCU applied (Fig.9). While minimum N plants uptake (29.08 kg ha^-1^) recorded where lower N applied with simple urea due to more losses. Maximum total N% in the soil after wheat crop harvesting showed by N 130 kg ha^-1^ BSCU in the soil under arid environment (Fig.10). But the lowest quantities (0.031) were determined by using a lower N level 94 kg ha^-1^ urea in soils. The nitrate-nitrogen contents in soil were found minimum by using coated fertilizers especially SCU and BSCU. In our experiment, N 130 kg ha^-1^ BSCU and SCU showed a decrease of 71.12 and 51.53% vs monotypic urea with the same N application (Fig.10). Among all sources, BSCU and SCU performed better than ordinary urea and NCU in the case of nitrate-nitrogen analysis of the topsoil layer than others (0–30 cm). The maximum NHI (64.70%) was recorded in N 130 kg ha^-1^ BSCU, while the minimum (32.98%) was computed in the experimental plot where N 94 Kg ha^-1^ urea is applied (Fig. 11d).

**Fig. 12:**
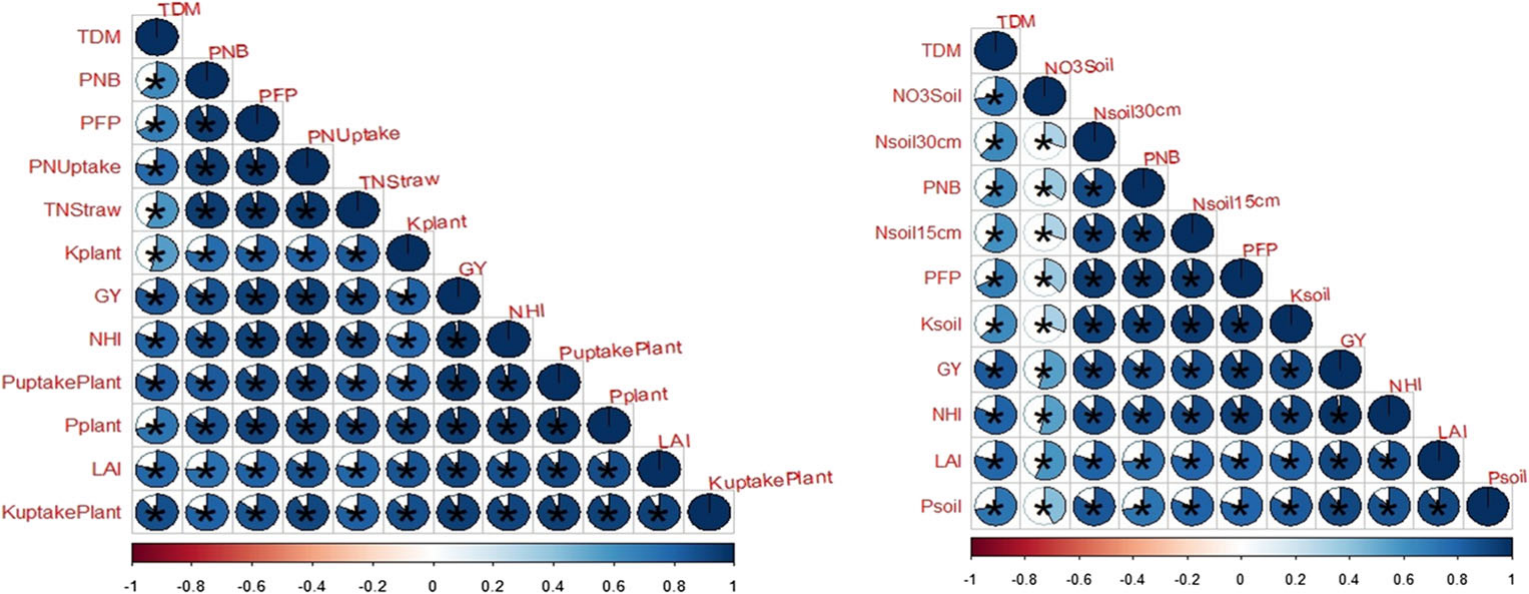
Correlation of different nitrogen use efficiency parameters of wheat and yield attributes. The areas of circles show the absolute value of corresponding correlation coefficients tested at *0.01 significance level. Dark blue and light blue color showed the higher and lower values. Star (*) showed the significance and non-star showed non-significance

It was seen that BSCU significantly increased PFP (Kg grain Kg^-1^ N supplied) to the extent of the N rate applied. Maximum PFP (43.85 Kg grain Kg^-1^ N supplied) was observed by 130 kg N ha^-1^, while minimum (19.893 Kg grain Kg^-1^ N supplied) computed by treatment N 94 kg ha^-1^ urea (Fig. 11b). According to the presented results, BSCU, SCU, and NCU significantly increased the PNB than monotypic urea fertilizer. The maximum PNB was found for higher N rates with source of BSCU treatment. Further, results declared that N 130 kg ha^-1^ BSCU improved PNB. Minimum PNB (0.190 Kg grain N content Kg^-1^ N supplied) showed by treatment N 94 kg ha^-1^ urea application in loamy soil under arid climatic conditions (Fig. 11a). NUE indices were influenced by different N rates. The optimum N rates have positive effects on wheat growth and yield attributes. The findings of the experiment presented a positive relationship between N rates and N use efficiency attributes like PFP, PNB, and NHI (%) (Fig. 12). The plants uptake maximum nitrogen when N is available in the topsoils for the maximum duration which leads to higher grain production and grain quality.

### P and K% in soil and plant uptake

The results showed that P and K have synergistic effects with coated N-based fertilizers. The maximum P and K (mg kg^-1^) in soil (0.34 and 1.31 (mg kg^-1^), respectively) were found with the application of 130 kg N ha^-1^ BSCU in the wheat plants. So, maximum P and K uptake (kg ha^-1^) is also showed by N 130 kg ha^-1^ BSCU as well, while the minimum P and K% in plants and uptake of both nutrients is showed by N 94 kg ha^-1^. The highest P (5.45 mg kg^-1^) was recorded in soil depth of 0–30 cm where N 130 kg ha^-1^ is applied, while the minimum (4.95 mg kg^-1^) P was recorded in monotypic urea with an N level of 94 kg ha^-1^ application. The K in soil was also recorded highest in coated urea-treated plots. The maximum and minimum K (100.67 and 85.67 mg kg^-1^) were recorded in plots where N 130 kg ha^-1^ BSCU and N 94 kg ha^-1^ urea are applied, respectively (Table 3). The N showed synergistic effects with P and K application in the soils. The optimum availability of N contents in soil showed a positive correlation with P and K uptake in wheat plants. The plants’ NPK contents % improved growth yield and yield attributes as well (Fig. 12 ).

**Table 3:** Effect of different N levels and slow-release urea fertilizers on the ratio of phosphorus and potassium in soil and plants under arid environmental conditions

Treatments	P (mg kg^-1^) in soil	K (mg kg^-1^) in soil	P% in plants	K% in plants	P uptake (Kg ha^-1^)	K uptake (Kg ha^-1^)
UreaN_0_	5.33 f	94.00 de	0.28 cd	1.06 c	39.70 b	177.67 e
UreaN1	5.30 g	91.33 gh	0.25 e	1.05 c	33.26 d	158.55 h
UreaN2	4.99 k	88.67 j	0.20 f	1.02 cde	23.23 fg	127.30 k
UreaN3	4.95 l	85.67 k	0.16 g	0.85 f	16.18 h	103.82 o
NCUN_0_	5.38 d	95.33 cd	0.29 bc	1.07 bc	41.32 ab	186.72 c
NCUN1	5.36 e	92.67 efg	0.26 de	1.08 bc	34.08 d	162.58 g
NCUN2	5.26 h	90.67 hi	0.20 f	1.05 c	22.88 fg	129.18 k
NCUN3	5.00 k	89.33 ij	0.18 g	0.89 ef	18.37 h	110.91 n
SCUN_0_	5.39 cd	97.67 b	0.31 b	1.20 ab	41.14 ab	194.44 b
SCUN1	5.40 bc	93.33 ef	0.29 bc	1.20 ab	35.89 cd	174.26 f
SCUN2	5.27 h	91.67 fgh	0.22 f	1.03 cd	25.67 ef	140.58 j
SCUN3	5.04 j	90.33 hij	0.20 f	0.91 def	22.10 g	115.23 m
BSCUN_0_	5.45 a	100.67 a	0.34 a	1.31 a	43.83 a	205.36 a
BSCUN1	5.30 b	96.33 bc	0.31 b	1.25 a	38.49 bc	181.67 d
BSCUN2	5.41 g	92.67 efg	0.24 e	1.09 bc	28.32 e	144.87 i
BSCU4N3	5.07 i	91.33 gh	0.21 f	0.98 cde	21.99 g	121.59 l

*NCU* neem-coated urea, *SCU* sulfur-coated urea, *BSCU* bioactive sulfur-coated urea

*N*_*0*_= 130 kgha^-1^; *N1*= 117 kgha^-1^; *N2*= 104 kgha^-1^; *N3*= 94 kgha^-1^

## Discussion

In the current experiment, plant response varied to coated urea fertilizers under arid environmental conditions. This is an interaction between different N levels and coated fertilizer addition. The wheat crop showed an effective response to coated fertilizers with an N level of 130 kg ha^-1^ application as compared with monotypic urea. The N fertilizers showed non-effective results on days to seedling emergence, and it was consulted to the results of Khan et al. (2008) which showed days to emergence were not affected by N and tillage in the first year of planting but improved in the next following year due to high availability of nutrients and water availability in the soil. Our results showed that due to the use of coated fertilizers, crop reached anthesis and maturity phases early.

The experiment showed that various N levels and coated urea (NCU, SCU) attained maximum LAI, and findings are agreed with Shivay et al. (2016). Shivay et al. (2015) and Joshi et al. (2014) also showed that maximum N level with NCU and SCU enhanced LAI as compared with simple urea because of minimum leaching and volatilization losses (Chaudhari et al. 2006). Gudge et al. (2019) showed that NCU and SCU showed an increase in LAI, spade value, TDM, 1000-grain weight, and harvest index of maize crop both years (2016–2018) as compared with prilled urea. Among coated urea fertilizers NCU performed best as compared with SCU in alkaline nature soil. Shivay et al. (2016) showed 5% SCU increased LAI up to 4.85 of spring wheat as compared with other coated treatments. Higher LAD was recorded where optimum N level and coated urea fertilizers were applied. Ullah et al. (2015) revealed that higher LAD was showed by higher N level fertilization. These findings were similar to the results obtained by Ullah et al. (2013) which showed N 210 kg ha^-1^ application significantly enhanced LAD. The maximum N level with coated urea enhanced mean CGR (g m^-2^ day^-1^). The current experiment showed that wheat crop plants attained higher CGR where N 130 kg ha^-1^ urea was applied. The number of tillers, LAI, and pre-anthesis CGR was increased with increment in N levels. The number of tillers of wheat was enhanced by the coating of urea fertilizers. These findings are in accord with other studies like Khandey et al. (2017) which found that treatment 125% NCU performed significantly better among other treatments for all the agronomic yield variables (number of panicle 345, number of tillers 323, panicle length 23.3 cm, the test weight of 1000 seeds of rice 28.6gm, straw 67 q ha^-1^ and grain 42.5 q ha^-1^, nutrient uptake 103 kg ha^-1^ N, 18.8 kg ha^-1^ P and 190 kg ha^-1^ K) in Vertisol. The LAI, LAD, and CGR showed a significant correlation with grains per spike, grain yield, and harvest index.

The findings are consulted to the results of Zheng et al. (2017) and Suganya et al. (2007), those found higher grain yield with the application of control released and NCU fertilization. The addition of SCU improved grain yield effectively by reducing rhizosphere pH and results tied with previous results found by Shivay et al. (2015). Guggari (2018) showed that NCU recorded significantly higher grain yield (2965 kg ha^-1^), grain weight (41.4 g), and stover yield (6.13 t ha^-1^) compared with other types of urea in pearl millet under rainfed condition. Our results are also revealed that BSCU and SCU at levels of N showed an increase in grain yield, number of grains per spike, grain weight, and harvest index. The 4–5% SCU showed higher grain yield of 9.58–11.21% and N uptake 19.06 to 23.94% vs 1,2,3% SCU and prilled urea in spring wheat (Shivay et al. 2016 ). The coated urea fertilizer application with recommended N rate presented higher seeds per spike and other yield attributes The impact of various N rates through NCU and SCU fertilization on wheat crop was in line with other researcher’s results presented in various scientific studies (Shivay et al. 2016; Kumar et al. 2015). The findings showed that maximum N levels and coating of urea fertilizer with neem oil and elemental sulfur significantly enhanced total dry matter of cereal crops and accord with results presented by different researchers (Ghobadi et al. 2010; Hayat and Khan 2013; and Iqbal et al. 2013). Our findings were confirmative with Ullah et al. (2018) and Pushpanathan et al. (2005), which showed that coated fertilizer and higher N rate significantly enhanced thousand-grain weight. The results further depicted that different levels of coated N fertilizers affected the wheat harvest index considerably. The results are in line with Laghari et al. (2010) who observed a higher harvest index by using recommended N level application. Shivay et al. (2016) showed that HI increased 4% SCU than monotypic urea and other S concentration coating on granular urea fertilizer. It is consulted with results of Sarwar et al. (2019) which showed that maximum N levels with a blend of bio-fertilizer and neem seeds extract increased harvest index of wheat. The results are confirmed with a study organized by Joshi et al. (2014) who showed NCU increased chlorophyll contents up to 42.05 (SPAD) of corn. Results are confined with Zong et al. (2010) who found that SCU significantly enhanced the maize chlorophyll concentration and grain yield. Optimum N level fertilization as coated urea increased wheat net photosynthetic rate. Our findings were confirmed with Ali et al. (2007) who showed that treatments containing 100 and 75% dose of neem oil coated urea showed an increase of 39.01 and 14.82% in photosynthetic rate, respectively, as compared with prilled urea in maize crop under alkaline calcareous soil. The chlorophyll contents and net photosynthetic rate also showed a highly significant correlation with grain yield.

The soil total N% enhanced because of N availability for a long-term period by using coated materials. The earlier experiment showed that control released fertilizers were enhanced total N% with equal N level application vs monotypic urea in the soil (Zheng et al. 2016). The findings were in line with Gangurde et al. (2018) who found NCU (100%) application enhanced soil available N (188.40 kg ha^-1^), P (18.10 kg ha^-1^), and K (490.63 kg ha^-1^) contents significantly in pearl millet crop. Our findings were confirmed with evidence showed by Ali et al. (2007) who showed that neem oil-coated urea reduced nitrate losses due to neem nitrification inhibitors and higher nitrate contents indicated by commercial urea fertilizer. Furthermore, maximum nitrate concentration is observed with minimum coating concentration at 50% NCU and decreased with the application of 100% NCU in maize as compared with monotypic urea. The positive effects of different coated urea fertilizers on NUE have been observed in the current experiment and confirmative with studies showed by Meena and Shivay (2010) and Bana and Shivay (2012). Shivay et al. (2016) also presented that 5% SCU increased wheat crop PFP 36.6 (Kg grain Kg^-1^ N applied) and NHI 73.3% as compared with a non-coated and lower concentration of S coatings. The results were in accord with Thind et al. (2010) who found NCU benefits over monotypic urea and improved NUEs and plant’s N uptake. In the current experiment, coated fertilizers BSCU and SCU with N rate 130 kg ha^-1^ showed the highest NUE components as PFP (Kg grain Kg^-1^ N supplied) and PNB (Kg grain N content Kg^-1^ N supplied) as compared with NCU and urea. The soil N availability showed a significant correlation with NUEs like PFP (Kg grain Kg^-1^ N supplied) and PNB PNB (Kg grain N content Kg^-1^ N supplied). The N has synergistic effects with P and K nutrients, so, our correlation results also showed significant results. The plant NPK uptake showed a significant correlation with grain yield. The plant N uptake showed highly significant results with NHI%. Zarei et al. (2013) and Lu et al. (2015) revealed that correlation showed a positive relation between grain’s nitrogen concentrations and yield (Zarei et al. 2013; Lu et al. 2015).

## Conclusion

Different strategies like slow-release fertilizers are used to increase NUE of nitrogen-based fertilizers in arid agriculture regions. Respectively, the environmental hazard of ammonia volatilization and nitrate leaching decreased effectively by using coated urea fertilizers. The current experiment showed the positive effects of coated fertilizers on wheat growth and development, physiological, yield, and NUEs. The coated urea fertilizers inhibited nitrification and volatilization processes and increased N availability in the soil for plant consumption. A significant increase in wheat growth and development was shown with BSCU when applied at N recommended rate (130 kg ha^-1^). The same N level 130 kg ha^-1^ showed an increase of 5.17% in LAI as compared with simple urea. The number of tillers, 1000-grain weight, and harvest index showed an increase of 10, 31, and 21.87% vs urea with nitrogen 130 kg ha^-1^ application than simple urea. The bioactive sulfur-coated urea with an N rate of 130 kg ha^-1^ showed an increase of 10% in grain yield vs monotypic urea. The S-coated urea fertilizer enhanced plant and soil nitrogen contents as compared with neem oil-coated urea. The S and microbes coated urea reduced rhizosphere pH and showed effective results in calcareous soils under arid climatic conditions. It was observed that bioactive sulfur-coated urea and sulfur-coated urea performed best than neem oil-coated urea and simple urea. The bioactive sulfur-coated urea with nitrogen 130 kg ha^-1^ showed an increase of 22.17% in plant nitrogen uptake and a decrease in nitrate concentration as compared with simple urea. The coated urea fertilizers improved phosphorus and potassium uptake in wheat crop due to the synergistic effect of nitrogen with phosphorus and potassium. The urea fertilizers coated with secondary minerals like S improved the nitrogen release ability of urea that fulfills the plants’ requirement of nitrogen. The 80 and 90% N rates with coated urea especially with S and bioactive S increased all growth, yield, and nitrogen-related components of wheat under arid environmental conditions. So, lower N (117 and 104 kg ha^-1^) with sulfur and bioactive sulfur-coated urea may also be recommend for wheat growers in the region to minimize the N losses and sustainability of the ecosystem. Future research studies like the modeling of ecosystem services and N loss under various crops and climate change scenarios may also suggest the sustainability of the agricultural system.

## Data Availability

The datasets used and/or analyzed during the current study are available from the corresponding author on reasonable request.
